# The genetics of feto-placental development: A study of acid phosphatase locus 1 and adenosine deaminase polymorphisms in a consecutive series of newborn infants

**DOI:** 10.1186/1477-7827-6-38

**Published:** 2008-09-03

**Authors:** Fulvia Gloria-Bottini, Adalgisa Pietropolli, Luca Coppeta, Andrea Magrini, Antonio Bergamaschi, Egidio Bottini

**Affiliations:** 1 Division of Human Population Biopathology and Environmental Pathology, Department of Biopathology and Imaging Diagnostics, University of Rome Tor Vergata, Rome, Italy; 2 Division of Obstetrics and Gynecology, Department of Surgery, University of Rome Tor Vergata, School of Medicine, Rome, Italy; 3 Institute of Occupational Health Medicine, Catholic University of Holy Hearth, Rome, Italy

## Abstract

**Background:**

Acid phosphatase locus 1 and adenosine deaminase locus 1 polymorphisms show cooperative effects on glucose metabolism and immunological functions. The recent observation of cooperation between the two systems on susceptibility to repeated spontaneous miscarriage prompted us to search for possible interactional effects between these genes and the correlation between birth weight and placental weight. Deviation from a balanced development of the feto-placental unit has been found to be associated with perinatal morbidity and mortality and with cardiovascular diseases in adulthood.

**Methods:**

We examined 400 consecutive newborns from the Caucasian population of Rome. Birth weight, placental weight, and gestational length were registered. Acid phosphatase locus 1 and adenosine deaminase locus 1 phenotypes were determined by starch gel electrophoresis and correlation analysis was performed by SPSS programs. Informed verbal consent to participate in the study was obtained from the mothers.

**Results:**

Highly significant differences in birth weight-placental weight correlations were observed among acid phosphatase locus 1 phenotypes (p = 0.005). The correlation between birth weight and placental weight was markedly elevated in subjects carrying acid phosphatase locus 1 phenotypes with medium-low F isoform concentration (A, CA and CB phenotypes) compared to those carrying acid phosphatase locus 1 phenotypes with medium-high F isoform concentration (BA and B phenotypes) (p = 0.002). Environmental and developmental variables were found to exert a significant effect on birth weight-placental weight correlation in subjects with medium-high F isoform concentrations, but only a marginal effect was observed in those with medium-low F isoform concentrations. The correlation between birth weight and placental weight is higher among carriers of the adenosine deaminase locus 1 allele*2, which is associated with low activity, than in homozygous adenosine deaminase locus 1 phenotype 1 carriers (p = 0.04). The two systems show a cooperative effect on the correlation between birth weight and placental weight: the highest value is observed in newborns carrying adenosine deaminase locus 1 allele*2 and acid phosphatase locus 1 phenotypes with medium-low F isoform concentration (p = 0.005).

**Conclusion:**

These data suggest that zygotes with low adenosine deaminase locus 1 activity and low F activity may experience the most favourable intrauterine conditions for a balanced development of the feto-placental unit.

## Background

We have recently described a cooperative interaction between ACP_1 _(acid phosphatase locus 1) and ADA_1 _(adenosine deaminase locus 1) genetic polymorphisms concerning their effects on the susceptibility to spontaneous primary repeated miscarriages: women carrying the ADA_1_*2 and ACP_1_*C alleles show the lowest susceptibility to repeated miscarriages [1]. Both systems share important effects on glucose metabolism and immunological function. These observations prompted us to search for a possible cooperative interaction between the two systems regarding their effects on developmental parameters during intrauterine life.

It is likely that a balanced growth of the two portions of the feto-placental unit (i.e. without the prevalence of placental on the fetal part or vice versa) represents an advantage for fetal development. The birth weight/placental weight ratio (BW/PW) has been found to be correlated with perinatal morbidity and mortality and with cardiovascular disease in adulthood [2,3]. There is evidence that in addition to maternal factors and socioeconomic status, genetic factors also influence the ratio BW/PW [3-5]. In a recent note we have proposed the correlation between BW and PW as an index of balanced development of the feto-placental unit and have shown that this correlation is influenced by ACP_1 _phenotype [6].

### The ACP_1 _genetic polymorphism

ACP_1 _also called low molecular weight phospho-protein tyrosine phosphatase (LMPTP) is an enzyme controlled by a locus on chromosome 2 showing three common alleles: ACP_1_*A, ACP_1_*B, ACP_1_*C. These three alleles are associated with different enzymatic activities [7]. Activity of ACP_1 _phenotypes are in te following order: A < BA < B = CA < CB < C [8].

Each allele at the ACP_1 _locus encodes two isoforms, called F (fast) and S (slow) [9,10]. ACP_1 _B and BA show a medium-high F isoform activity while CB, A, CA and C phenotypes show a medium-low F isoform activity. ACP_1 _C, CA and CB show a much higher activity of S isoform as compared to other ACP_1 _phenotypes [7,10].

Two important functions have been suggested for ACP_1_: flavin-mono-nucleotide phosphatase activity and tyrosine phosphatase activity [11-13]. Catalysing the conversion of flavin-mononucleotide (FMN) to riboflavin, ACP_1 _may have a role in regulating the cellular concentration of flavin-adenine-dinucleotide (FAD), flavo-enzyme activity and energy metabolism. As a phosphotyrosine phosphatase, the enzyme may have an important role in cellular growth regulation and in modulation of glycolytic rate through the control of receptor activities and of band 3 protein phosphorylation status [[12,14] and [15]].

Recently it has been shown that ACP_1 _specifically dephosphorylates the negative regulatory Tyr-292 of ZAP-70, thereby counteracting inactivation of ZAP-70. The ZAP-70 protein-tyrosine kinase plays a central role in signalling from the T cell receptor. Thus, these results indicate that ACP_1 _strengthens T cell receptor signalling [16].

### The ADA_1 _genetic polymorphism

ADA_1 _is a polymorphic enzyme present in all mammalian tissues [17]. It is controlled by a locus with two codominant alleles ADA*1 and ADA*2 located on the long arm of chromosome 20. The corresponding three common ADA_1 _phenotypes have different enzymatic activities: the ADA_1_1 phenotype is 15% more active than the ADA_1_2/1 phenotype and 30% more active than the ADA_1_2 phenotype, which is very rare [18].

ADA_1 _catalyses the irreversible deamination of adenosine to inosine. Red Blood Cells (RBC) are in equilibrium with freely diffusing adenosine [19], pointing to an important role for this enzyme in the regulation of adenosine concentration.

Current interest has been focused on a wide variety of effects produced by adenosine via activation of cell surface adenosine receptors [20,21]. Adenosine counteracts insulin action in the liver by activating A2B receptors [22]. Adenosine seems to facilitate insulin action in adipocytes.

The adenosine deaminase complex protein [23] (ADPC) is identical with CD26, a T cell activating antigen and with a glycoprotein present in epithelial cells of various tissues. Recent data suggest that ADA_1 _and CD26 are co-localized on the T cell surface but not inside cells.

Cells expressing ADA_1 _and CD26 on the surface are much more resistant to the inhibitory effects of adenosine. These data suggest that ADA_1 _on the cell surface is involved in an important immunoregulatory mechanism by which released ADA_1 _binds to the cell surface of CD26, and this complex is capable of reducing the local concentration of adenosine [24].

In the present paper we have performed a more detailed analysis of the effect of ACP_1 _polymorphism and have extended this study to the ADA_1 _polymorphism. On the basis of the observations on women with repeated spontaneous miscarriages we would expect an optimal developmental context in zygotes carrying the ADA_1_*2 and ACP_1_*C alleles.

## Methods

In the present study we examined 400 consecutive newborn infants from healthy puerperae. All infants were Caucasian from the population of Rome. Birth weight and placental weight (wet, untrimmed) were registered in the delivery room. Gestational length was estimated from the date of the last menstrual period and checked against Dubowitz score as an additional index of neonatal maturity. Multiples were excluded. Mode of delivery does not alter the phenotype of enzyme considered.

The data presented in the paper were collected a few years ago and at that time there was not an established Ethical Committee. The project was discussed and approved in the Department. Informed verbal consent to participate in the study was obtained from the mothers. This has been recently (April 28, 2008) approved by the Institutional Ethical Committee.

Newborn blood samples were obtained from the placental side of the umbilical vein after umbilical cord section. The ACP_1 _phenotype was determined in 361 newborns by starch gel electrophoresis on red blood cell hemolysates according to Harris and Hopkinson [25]. The acid phosphatase pattern is revealed by a solution of phenolphtalein diphosphate: the addition of ammonium solution reveals the area where phenolphtalein has been liberated in the areas of gel where ACP_1 _activity is present. In European populations the presence of three common alleles *A, *B and *C determines the occurrence of six phenotypes: A, AB, B, AC, BC and C. Each of the homozygous A, B and C phenotypes are composed of two fractions, F and S, corresponding to fast and slow components of the electrophoretic pattern. Heterozygous phenotypes have a pattern corresponding to a mixture of homozygous types.

The ADA_1 _phenotype was determined by starch gel electrophoresis on red blood cell hemolysates according to Spencer et al [26]. Inosine produced at the sites of ADA_1 _activity is converted in hypoxanthine in the presence of nucleoside phosphorylase and phosphate. The hypoxanthine is then oxidized by the action of xanthine oxidase, and during this reaction the tetrazolium salt MTT is reduced in the presence of phenazine methosulphate to a blue insoluble formazan. In the ADA_1_1 type there are three regularly spaced components which exhibit decreasing staining intensity in order of their anodal electrophoretic mobilities. In the ADA_1_2 type there are also three isozymes and their relative intensities and relative electrophoretic mobilities are very similar to those of the ADA_1_1 pattern. The difference between ADA_1_1 and ADA_1_2 is that the ADA_1_2 pattern is appreciably slower than the ADA_1_1 pattern. The pattern exhibiting four isozymes, designated ADA_1_2/1, has the appearance of a mixture of ADA_1_1 and ADA_1_2 patterns.

In the last few years, in our laboratory, determination of ADA_1 _and ACP_1 _genotypes has been performed routinely on DNA. In our laboratory the comparison of classical with DNA methods has shown practically no differences between phenotypic and genotypic classifications. On a sample of 50 subjects in which ACP_1 _and ADA_1 _phenotypes were determined by DNA and classical methods only one difference was observed for ACP_1 _and no difference for ADA_1_.

Correlation analysis was performed by SPSS programs. Differences between correlation coefficients were evaluated according to Snedecor and Cochran [27]. The distribution of ACP_1 _phenotypes among newborns does not differ statistically from Hardy-Weinberg expectation.

## Results

Table 1 shows demographic parameters of the sample study. Table 2 shows the distribution of ACP_1 _phenotypes and developmental parameters for each phenotype. No statistical significant difference among ACP_1 _phenotypes is observed for BW, PW and gestational duration. CA phenotype shows low values for all parameters, but these values are not statistically different from those of other ACP_1 _phenotypes. BW-PW correlation analysis shows highly significant differences among ACP_1 _phenotypes. The highest correlation coefficient is observed for CA phenotype and the lowest for B phenotype.

**Table 1:** Maternal and neonatal parameters in the sample study

	Mean	Proportion	S.E.
Maternal Age (yrs)	28.5		0.3
Gestational Age (wks)	39.6		0.12
Birth Weight (gr)	3269		29
Placental Weight (gr)	578		8
Smokers		38%	
Male Infant		54%	

**Figure 1:**
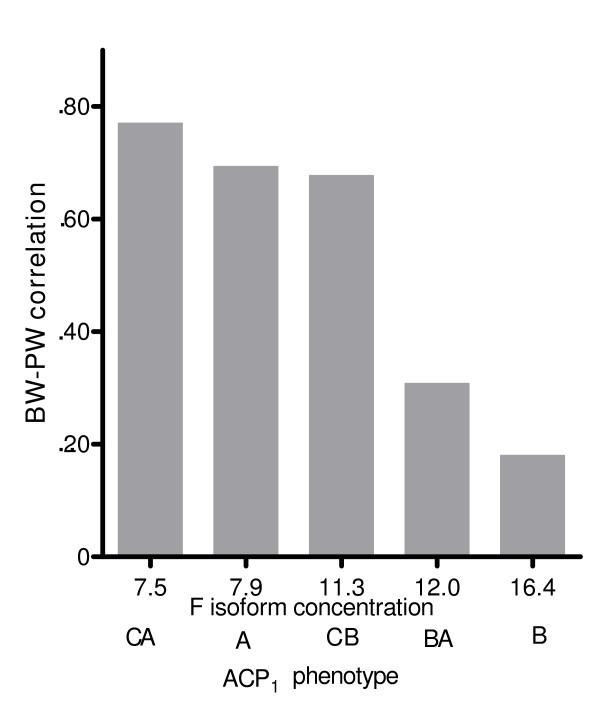
**The relationship between F isoform concentration and BW-PW correlation**. The term BW-PW correlation expresses the correlation between birth weight and placental weight. ACP1 is the acid phosphatase locus 1. A, B, C, BA, CA, CB are the ACP_1 _phenotypes. In abscissa F isoform concentrations of each ACP_1 _phenotype are also reported. The rank correlation coefficient according to Spearman (27) between BW-PW correlation and F isoform concentration is r_s _= -1, p < 0.01.

**Figure 2:**
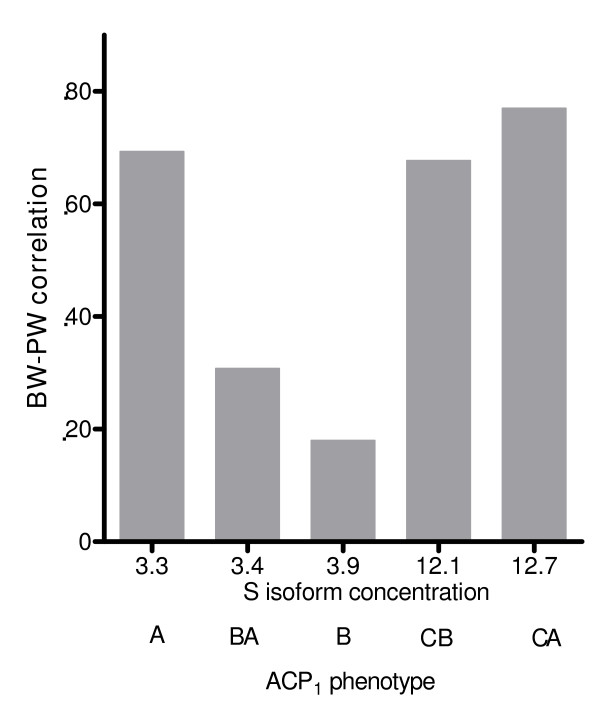
**The relationship between S isoform concentration and BW-PW correlation**. The term BW-PW correlation expresses the correlation between birth weight and placental weight. ACP1 is the acid phosphatase locus 1. A, B, C, BA, CA, CB are the ACP_1 _phenotypes. In abscissa S isoform concentrations of each ACP_1 _phenotype are also reported. The rank correlation coefficient according to Spearman (27) between BW-PW correlation and S isoform concentration is r_s _= 0.3, p not significant.

**Figure 3:**
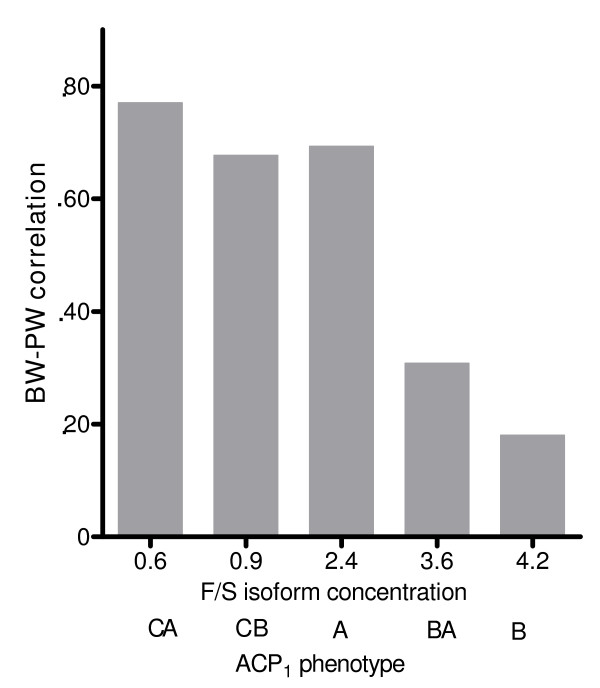
**The relationship between F/S isoform concentration and BW-PW correlation**. The term BW-PW correlation expresses the correlation between birth weight and placental weight. ACP1 is the acid phosphatase locus 1. A, B, C, BA, CA, CB are the ACP_1 _phenotypes. In abscissa F/S isoform concentrations of each ACP_1 _phenotype are also reported. The rank correlation coefficient according to Spearman (27) between BW-PW correlation and F/S isoform concentration is r_s _= -0.9, p < 0.05.

**Figure 4:**
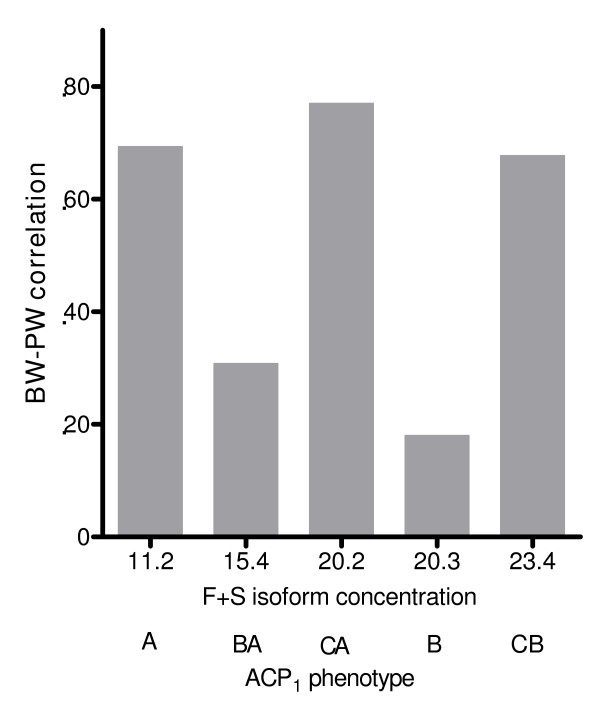
**The relationship between F+S isoform concentration and BW-PW correlation**. The term BW-PW correlation expresses the correlation between birth weight and placental weight. ACP1 is the acid phosphatase locus 1. A, B, C, BA, CA, CB are the ACP_1 _phenotypes. In abscissa F+S isoform concentrations of each ACP_1 _phenotype are also reported. The rank correlation coefficient according to Spearman (27) between BW-PW correlation and F+S isoform concentration is r_s _= 0.3, p not significant.

Figures 1, 2, 3, and 4 illustrate the relationship between BW-PW correlation and relevant ACP_1 _parameters: F and S activity, F/S activity ratio and total activity. The BW-PW correlation is negatively associated with F concentration (Fig 1) and F/S ratio (Fig 3). A, CA and CB phenotypes that share a medium-low F activity and F/S ratio have a high BW-PW correlation, while the B phenotype, which has the highest F activity and F/S ratio, has a low BW-PW correlation. No association is observed for S isoform concentration (Fig 2) and ACP_1 _total activity (Fig 4).

**Table 2:** Distribution of neonatal parameters in relation to ACP_1 _phenotypes.

	ACP_1 _phenotypes						Significance of difference among phenotypes (p)
		
	A	B	C	BA	CA	CB	
	
Absolute frequencies	34	163	2	123	9	30	
	
Percent frequencies	9.4%	45.2%	0.6%	34.1%	2.5%	8.3%	
*Birth weight (gr)*							

Mean	3345	3250	3645	3338	2837	3244	0.141 (*)
S.E.	85	46	575	44	333	90	

*Placental weight(gr)*							

Mean	590	594	615	571	517	540	0.491(*)
S.E.	23	17	135	12	46	21	

*Gestational age(wks)*							

Mean	40.00	39.61	39.50	39.77	38.11	39.53	0.405(*)
S.E.	0.41	0.20	1.50	0.15	1.57	0.40	
Median	40.00	40.00	39.50	40.00	40.00	40.00	

Correlation between birth weight and placental weight (r)	0.69	0.18	-	0.31	0.77	0.68	0.005(**)
Significance of r (p)	0.001	0.029	-	0.001	0.025	0.001	

Significance of differences among means (*) refers to Variance analysis. Significance of differences among correlation coefficients (**) has been calculated according to Snedecor and Cochran.

Table 3 shows the effect of smoking, maternal age, gestational age, parity and gender on the relationship between ACP_1 _and BW-PW correlation. For this analysis two classes of ACP_1 _phenotypes have been considered: A, CA, CB phenotypes with medium-low F activity, and B and BA phenotypes with medium-high F activity. In A, CA, CB phenotypes only maternal age exhibits some effect on the BW-PW correlation, while among B and BA phenotypes most variables show highly significant effects on the BW-PW correlation.

**Table 3:** Correlation between birth weight and placental weight in relation to ACP_1 _F isoform activity.

	ACP_1 _F isoform activity			
Sample	Medium-high (B+BA)		Medium-low (A+CA+CB)	
	
	r	p*	r	p*

*All subjects*	0.209	0.001	0.687	0.000

*Smoking*				

Yes	0.122	0.243	0.696	0.000
no	0.241	0.002	0.710	0.000
Significance of difference p**	0.000		0.750	

*Maternal age (yrs)*				

≤28	0.027	0.745	0.723	0.000
>28	0.478	0.000	0.640	0.000
Significance of difference p**	0.000		0.025	

*Gestational age (wks)*				

≤37	0.183	0.440	0.731	0.160
>37	0.228	0.000	0.655	0.000
Significance of difference p**	0.450		0.950	

*Birth order*				

1	0.305	0.001	0.672	0.000
≥ 2	0.158	0.060	0.703	0.000
Significance of difference p**	0.000		0.350	

*Sex*				

male	0.171	0.044	0.712	0.000
female	0.301	0.001	0.655	0.000
Significance of difference p**	0.000		0.170	

Significance of difference between correlation coefficients has been calculated according to Snedecor and Cochran. p* refers to significance of correlation coefficient. p** refers to significance of difference between the two classes. For "All subjects" the difference of correlation coefficients between (B+BA) vs (A+CA+CB) i.e. 0.209 vs 0.687 is highly significant: p = 0.002.

Table 4 shows the distribution of ADA_1 _phenotypes and developmental parameters for the ADA_1_1 phenotype and for carriers of the ADA_1_*2 allele. The BW-PW correlation coefficient is higher among carriers of the ADA_1_*2 allele than among ADA_1_1 phenotype carriers (p = 0.04). No significant difference between ADA_1 _phenotypes has been observed for BW, PW and gestational duration.

**Table 4:** Parameters distribution of newborns in relation to ADA_1 _phenotypes.

	ADA_1 _phenotypes		Significance of difference between phenotypes (p)
		
	ADA_1_1	(ADA_1_2/1+ADA_1_2)	
	
Absolute frequencies	317	58	
	
Percent frequencies	84.5%	15.5%	
*Birth weight (gr)*			

Mean	3279	3311	0.612*
S.E.	32	54	

*Placental weight (gr)*			

Mean	584	545	0.103*
S.E.	10	13	

*Gestational age (wks)*			

Mean	39.63	39.84	0.505*
S.E.	0.13	0.24	
Median	40.00	40.00	

Correlation between birth weight and placental weight (r)	0.289	0.552	0.040**
Significance of r (p)	0.000	0.000	

Significance of difference between means (*) refers to Variance analysis. Significance of difference between correlation coefficients (**) has been calculated according to Snedecor and Cochran

Table 5 shows the effect of smoking, maternal age, gestational age, parity and gender on the relationship between ADA_1 _and BW-PW correlation. Significant effects of these variables were observed in the correlations of BW-PW in both ADA_1 _subjects and in carriers of the ADA_1_*2 allele.

**Table 5:** Correlation between birth weight and placental weight in relation to ADA_1 _phenotypes.

	ADA_1 _phenotypes			
	
Sample	ADA_1_1		(ADA_1_2/1+ADA_1_2)	
	
	r	p*	r	p*
*All subjects*	0.289	0.000	0.552	0.000

*Smoking*				

Yes	0.275	0.003	0.484	0.036
no	0.288	0.000	0.589	0.000
Significance of difference p**	0.300		0.150	

*Maternal age (yrs)*				

≤28	0.104	0.181	0.679	0.000
>28	0.559	0.000	0.341	0.120
Significance of difference p**	0.000		0.000	

*Gestational age (wks)*				

≤37	0.292	0.187	0.922	0.078
>37	0.286	0.000	0.560	0.000
Significance of difference p**	0.900		0.350	

*Birth order*				

1	0.449	0.000	0.572	0.001
≥ 2	0.212	0.009	0.543	0.003
Significance of difference p**	0.000		0.700	

*Sex*				

male	0.268	0.000	0.359	0.078
females	0.344	0.000	0.692	0.000
Significance of difference p**	0.000		0.000	

Significance of difference between correlation coefficients has been calculated according to Snedecor and Cochran. p* refers to significance of correlation coefficient. p** refers to significance of difference between the two classes. For "All subjects" the difference of correlation coefficients between ADA_1_1 and (ADA_1_2/1+ADA_1_2) i.e. 0.289 vs 0.552 is statistically significant: p = 0.04.

The BW-PW correlation is influenced by environmental and developmental variables, but as shown in tables 3 and 5 the differential effects of ACP_1 _and ADA_1 _phenotypes on this correlation do not appear in general to be significantly influenced by these variables.

Table 6 shows the correlation between BW and PW according to the joint ACP_1_-ADA_1 _phenotype. Both ADA_1 _and ACP_1 _phenotypes were determined in 327 infants. There is a highly significant difference among joint phenotypes (p = 0.000): the highest correlation is observed in subjects who carry A or CA or CB phenotypes and the ADA_1_*2 allele, while the lowest correlation is observed when the ADA_1 _1 phenotype is associated with B or BA phenotypes.

**Table 6:** Correlation between birth weight (BW) and placental weight (PW) according to the joint ACP_1_-ADA_1 _phenotype.

	Joint ACP_1_-ADA_1 _phenotype			
Presence of ADA*2 allele	-	-	+	+
Presence of A or CA or CB types	-	+	-	+
BW-PW correlation coefficients (r)	0.193	0.613		0.648
Significance of r (p)	0.004	0.000		0.059
Total n°	217	101		9

Significance of difference among correlation coefficients p = 0.000. Significance of difference among correlation coefficients has been calculated according to Snedecor and Cochran

## Discussion and conclusion

The present data suggest that foetuses with low ADA_1 _activity, associated with medium-low ACP_1 _F isoform activity, have the best correlation between BW and PW, suggesting a most favourable situation for the development of the feto-placental unit. These observations agree with those expected on the basis of previous data on women with repeated spontaneous miscarriages that demonstrated a cooperative protective effect of ADA_1_*2 and ACP_1_*C alleles against fetal loss. Thus, the two lines of evidence support the hypothesis that foetuses with low ADA_1 _activity and low ACP_1 _F isoform activity have a balanced development of feto-placental unit and a higher probability of survival compared to other foetuses.

The exact mechanism underlying the statistical association of ADA_1 _and ACP_1 _with the BW-PW correlation is not known at present. An immunological mechanism is supported by the well known relationship between ADA_1 _and immune diseases and between ACP_1 _and T cell activation. A relative depression of T cell activation due to low level of F ACP_1 _isoform and to higher concentration of adenosine (due to the low activity of ADA_1_*2 carriers) could modulate the feto-maternal immunological relationship resulting in a balanced development of the two portions of feto-placental unit.

A metabolic mechanism may be operative in which ACP_1_, acting as phosphotyrosine phosphatase, could have an important role in the modulation of glycolytic rate through the control of insulin receptor activity and of band 3 protein phosphorylation status. Additionally, catalysing the conversion of flavin-mononucleotide (FMN) in riboflavin, the enzyme may influence flavo-enzyme activity and energy metabolism [7]. In turn, with respect to ADA_1 _activity, recent studies have shown that adenosine counteracts insulin action in the liver by activating A2B receptors [20-22]. On the basis of these actions on glucose metabolism, ACP_1 _F isoform activity coupled with low ADA_1 _activity could have favourable effects on the development of the feto-placental unit.

Regarding the effect of genetic variability of ACP_1 _on the correlation of BW with PW, it is interesting to speculate on the possible selective advantage of the *A and *C alleles over the *B allele. The *B allele is the most frequent in all human populations, and the *A allele is present with variable frequencies in all major ethnic groups, while *C allele is present with appreciable frequencies only in Caucasians.

Our data suggest that the optimal BW-PW correlation is seen in carriers of the ACP_1_*C allele and in the homozygous A phenotype (table 4 and Fig 1), while the heterozygous BA phenotype shows an intermediate value between B and A (Fig 1). Interestingly, in A, CA and CB phenotypes the BW-PW correlation is hardly influenced by environmental circumstances, while in B and BA phenotypes the environmental variables exert considerable effects on this correlation. Thus, ACP_1_*A and *C variants could have a selective advantage during intrauterine life on the fundamental ACP_1_*B allele. This might have contributed to an increase in frequencies of the ACP_1_*A and *C alleles to polymorphic values and could presently contribute to maintenance of the ACP_1 _polymorphism in human populations.

## Competing interests

The authors declare that they have no competing interests.

## Authors' contributions

GBF, BE, MA and BA have been involved in the conception and design of the study, in drafting the manuscript and in its critical revision. GBF and BE have interpreted the data and performed the statistical analyses. PA and CL have contributed to the revision of the manuscript, to the acquisition of the data and to coordination of study. All authors have read and approved the final manuscript.
